# The application of AI-assisted music therapy tools in mental health interventions

**DOI:** 10.3389/fpsyg.2026.1741463

**Published:** 2026-01-22

**Authors:** Qiuyan Wei, Wenting He

**Affiliations:** 1 Saint Petersburg State Institute of Culture, Saint Petersburg, Russia; 2 Zhengzhou University, Zhengzhou, China

**Keywords:** artificial intelligence, digital health, emotion regulation, intervention, mental health, music therapy

## Abstract

With the rising prevalence of mental health problems across populations, the limitations of traditional interventions have become more evident. Music therapy has received growing attention as a psychological intervention, and recent advances in artificial intelligence (AI) have created new opportunities for this field. This review examines the application potential and implementation pathways of AI-assisted music therapy tools for mental health interventions. Drawing on the literature and representative cases, this review summarizes application models and reported effects of AI in music therapy. The available evidence suggests that AI-assisted music therapy tools can support personalized interventions by adapting to users’ emotional and psychological states. Reported benefits include reductions in anxiety and depressive symptoms and improvements in emotion regulation across groups such as children, adolescents, and older adults. Finally, this review outlines priorities for future translation into mental health services and emphasizes data privacy and ethical standards to ensure responsible deployment.

## 1 Introduction

### 1.1 Research background

#### 1.1.1 Imbalance between supply and demand in mental health interventions

At present, the global mental health intervention system faces a severe challenge of “supply–demand imbalance.” Recent global estimates suggest, on average, only 2% of national health budgets are allocated to mental health (global average; based on cross-country spending estimates/compiled national health accounts; around 2020) (Hasnain et al., 2020). In low-income countries, there is only one mental health worker per 100,000 people (workforce density per 100,000 population; WHO-reported service resources, 2021), and essential psychotropic medications remain inaccessible or unaffordable for many (WHO service availability/affordability indicators, 2021) (World Health Organization [WHO], 2021). The digital divide further exacerbates service inequality. This shortage has led to low treatment coverage—only about 9% of patients with depression receive adequate treatment (defined as minimally adequate treatment; country-level modeled estimates for 2021)—and large disparities in service quality, with high rates of involuntary hospitalization and insufficient social support (Santomauro et al., 2024).

China is highlighted here because its large population and pronounced regional disparities in mental-health service accessibility coexist with rapid growth in digital and remote health delivery, making it a particularly relevant context for AI-enabled, scalable interventions. In China, although the number of certified psychological counselors has grown, the proportion of those with systematic professional training and clinical competence remains low (Yue et al., 2022). Moreover, social stigma, geographical barriers, and difficulty accessing appointments prevent many individuals with psychological distress from receiving timely intervention (Shi et al., 2020). These structural deficiencies have driven the field of mental health toward intelligent, remote, and self-guided modes of intervention.

These constraints highlight the need for scalable, accessible, and low-cost complementary interventions that can be delivered beyond specialist settings, which motivates interest in music-based approaches.

#### 1.1.2 The value and applicability of music therapy in mental health interventions

Mental health problems have become a major global public health challenge, with the prevalence of depressive and anxiety disorders continuing to rise (GBD 2019 Mental Disorders Collaborators, 2022). Although traditional pharmacological and psychotherapeutic treatments are effective, they often entail side effects and face limitations in accessibility and resource availability, prompting the search for safe and effective complementary approaches (Strawn et al., 2023).

As a non-pharmacological intervention, music therapy has shown promising outcomes in emotion regulation, stress reduction, and cognitive function enhancement (de Witte et al., 2022; Jiménez-Palomares et al., 2024; Lu and Song, 2025), though more rigorous clinical evidence is needed to support these findings. It provides a flexible, accessible, and humanistic alternative that integrates emotional expression, aesthetic experience, and therapeutic communication, making it particularly suitable for diverse populations in need of psychological support (Gustavson et al., 2021). A growing body of research has demonstrated that music can activate the brain’s reward system, stimulate the release of dopamine and reduce levels of the stress hormone cortisol, thereby alleviating symptoms of anxiety and depression (Choi et al., 2008).

For instance, a systematic review published in 2017 indicated that incorporating music therapy into standard treatment significantly improved depressive symptoms, showing greater efficacy compared to standard treatment alone (Geretsegger et al., 2017). A meta-analysis of 47 studies revealed that music therapy exerted a moderate-to-strong overall effect on reducing psychological stress (de Witte et al., 2022). Music therapy has also been shown to be widely applicable across different populations, though further controlled studies are needed to confirm these effects.

Among adolescents and college students, group-based music activities help promote active stress reduction and prevent anxiety (Finnerty and Trainor, 2025). For older adults with dementia, music therapy can improve mood and reduce behavioral problems (Geretsegger et al., 2017). For children with autism spectrum disorder (ASD), musical training enhances communication and social interaction skills (Lim, 2010). With its safety, noninvasiveness, and high acceptability among participants, music therapy is increasingly recognized as an important complementary approach in the field of mental health intervention.

However, delivering music therapy with consistent personalization and continuous outcome monitoring at scale remains challenging in traditional formats, which provides a clear rationale for integrating AI.

#### 1.1.3 Integration pathways and intervention advantages of AI technology in music therapy

In recent years, AI has developed rapidly and shown transformative potential in the field of healthcare. AI can efficiently analyze vast amounts of data, recognize complex patterns, and provide personalized decision support (Kaggwa et al., 2024). Integrating AI into the domain of music therapy offers the prospect of overcoming the limitations of traditional approaches and enhancing both precision and accessibility in psychological interventions. AI represents a radical change for music therapy because it shifts intervention delivery from largely therapist-driven, session-based decisions to data-driven, scalable, and continuously adaptive systems. By enabling multimodal sensing (e.g., affective and physiological signals) and closed-loop adjustment of music selection/generation in real time, AI transforms static “music prescriptions” into personalized interventions that can be deployed remotely and evaluated through continuously logged process and outcome data.

For example, AI systems can dynamically adjust music prescriptions in real time based on patients’ physiological and emotional feedback, thereby achieving personalized and adaptive therapy. Through the use of smartphones and other intelligent devices, individuals can access tailored music-based therapeutic experiences anytime and anywhere. This “AI + music therapy” model aligns with the broader trend of digital health development, providing an innovative solution to the shortage of professional therapists and promoting equitable access to mental health interventions (Sun et al., 2024).

More importantly, the combination of AI’s computational precision and music’s emotional resonance may produce a synergistic effect: AI contributes the scientific accuracy of diagnosis and response, while music offers the humanistic support of emotional engagement. The integration of these two dimensions holds the potential to foster more effective psychological recovery and well-being. Therefore, conducting systematic research on AI-assisted music therapy tools is not only of theoretical and academic significance but also of substantial clinical and social value.

Despite these advances, the central problem is that current work on AI-assisted music therapy remains fragmented: studies often mix “technology components” with “therapeutic delivery,” vary widely in target outcomes and evaluation rigor, and lack a clear framework linking AI functions (e.g., monitoring, personalization, generation) to specific therapeutic aims and evidence standards. Therefore, this review aims to synthesize existing research and representative tools, clarify key pathways through which AI can support music therapy, and identify critical gaps and priorities for rigorous validation and responsible implementation.

### 1.2 Existing evidence and gaps

Internationally, research on the application of AI in music therapy is still in its formative stage but has shown rapid growth and promising potential. Current work can be broadly grouped into several directions, including AI-driven music generation, neurobiological rationales for music-based effects, and biofeedback-based personalization. For example, Williams et al. developed an AI-based approach to functional music generation in which machine learning models composed music intended to induce specific emotional states, and musical parameters were adaptively adjusted in real time using electrodermal feedback (Williams et al., 2020). Related reviews have further discussed how musical stimulation may engage brain regions involved in emotion and cognition, and how combining music with AI-driven biofeedback could enhance personalization by adjusting musical parameters based on physiological responses (Jiao, 2025). Meanwhile, emerging technology platforms have begun to translate these ideas into early-stage products and services, suggesting increasing practical interest. However, the evidence base remains uneven, and many studies rely on preliminary or small-sample evaluations; more rigorous designs (e.g., randomized controlled trials and external validation) are needed to substantiate clinical efficacy and generalizability.

In China, interest in AI-assisted music therapy has also risen, supported by emerging cross-disciplinary collaborations between music therapy and AI. Representative initiatives include laboratory-based development of AI-generated sleep-aid music guided by physiological signals (e.g., EEG and heart rate) in pilot interventions. In addition, Wang et al. (2024) applied an optimized long short-term memory (LSTM) network to analyze process data from music therapy sessions and predict treatment outcomes in a trauma-related context, reporting approximately 85% predictive accuracy. Overall, these efforts suggest that localized integration of AI and music therapy is feasible and may support more personalized psychological services for specific populations. Nevertheless, domestic research remains at an early stage and is dominated by proof-of-concept studies and small-sample experiments, with limited large-scale implementation and insufficient methodological standardization.

Taken together, existing studies and applications remain fragmented and heterogeneous. A key gap is the lack of a coherent framework that separates and links AI functional components (e.g., sensing/monitoring, personalization, and generation) with therapeutic delivery models and target outcomes, alongside inconsistent evaluation metrics and limited use of rigorous comparative designs. Therefore, a systematic synthesis of theoretical foundations, technical frameworks, representative applications, and implementation challenges is needed to clarify what is currently supported by evidence and to define priorities for robust validation and responsible translation.

In this review, we address this gap by proposing a functional, closed-loop conceptual framework that organizes AI-assisted music therapy into four linked modules: (1) sensing and assessment, (2) personalization and adaptive control, (3) generative music approaches, and (4) deployment, logging, and remote support. This framework is used to structure the Results and Discussion and to connect AI components to therapeutic delivery models, target outcomes, and evidence standards.

### 1.3 Objective and review questions

The objective of this review is to synthesize and integrate current evidence on AI-assisted music therapy for mental health interventions, with the aim of clarifying its core components, intervention pathways, and implementation considerations across interdisciplinary contexts. Specifically, this review addresses the following questions: (1) What are the major AI functional components used in AI-assisted music therapy (e.g., emotion recognition, personalized recommendation, interactive feedback, and music generation), and how are they mapped onto the intervention logic of music therapy? (2) Across which populations and target outcomes (e.g., anxiety, depression, stress regulation, sleep, and well-being) have these tools been applied, and what evidence is currently available regarding effectiveness and feasibility? (3) What methodological limitations and evidence-quality issues characterize the existing literature (e.g., small samples, proof-of-concept designs, limited comparators, and lack of external validation)? (4) What practical barriers and risks (e.g., privacy/ethics, individual differences, and evaluation standardization) constrain real-world deployment, and what priorities can guide responsible translation into digital mental health services (e.g., mobile and wearable platforms)?

### 1.4 Conceptual framework

In this review, AI-assisted music therapy refers to music-based mental health interventions in which AI supports at least one core step of the therapeutic process, such as sensing/assessment, personalization/adaptive control, and/or generative music production. Personalized intervention denotes tailoring musical content and its adaptation rules to an individual’s current state, preferences, and therapeutic goals, and emotional regulation mechanisms refer to the processes through which music helps modulate affective intensity/arousal and coping responses, which we interpret using established emotion regulation, neuroscientific, and HCI perspectives.

#### 1.4.1 Emotion regulation theory

Emotion Regulation Theory provides a core psychological framework for understanding how music-based interventions support mental health by helping individuals modulate emotional intensity and quality through strategies such as attentional shifting and cognitive reappraisal. Music is frequently used intentionally to alter mood and relieve stress, and evidence suggests that appropriate music under stress can reduce tension and anxiety while enhancing positive affect (Lee et al., 2016; Knott and Block, 2020). Within AI-assisted music therapy, this framework highlights the importance of aligning music selection or generation with the user’s current emotional state, thereby supporting personalized regulation goals (e.g., calming music for anxiety and activating music for depressive mood states).

#### 1.4.2 Neuroscientific theory of music

Neuroscientific accounts explain how music engages widespread neural networks related to emotion, reward, attention, memory, and motor coordination, providing a biological rationale for therapeutic effects (Salimpoor et al., 2011; Särkämö et al., 2013). Koelsch’s “Five-Component Model” further proposes that music can modulate attention, emotion, cognition, behavior, and communication through distinct neuropsychological pathways (Koelsch, 2009). For AI-assisted music therapy, neuroscientific principles inform how systems can operationalize sensing/assessment (e.g., physiological and behavioral signals) and translate these signals into evidence-informed music selection and adaptive adjustment during interventions.

#### 1.4.3 Human–computer interaction theory

Human–Computer Interaction (HCI) Theory emphasizes user-centered design, usability, and feedback, which are essential for ensuring that AI-assisted music therapy tools remain acceptable, safe, and engaging for users. In this context, HCI principles support mechanisms that enhance engagement and adherence, such as intuitive interfaces, meaningful user control (e.g., preference inputs and self-reports), and responsive feedback loops (Caceres and Ghosh, 2022). Together with adaptive system principles, HCI provides a design rationale for closed-loop interventions in which user data continuously guide system outputs, allowing AI tools to learn user preferences and response patterns and to optimize music-based support over time (Holohan and Fiske, 2021; Shen et al., 2024).

Link to the review structure: Guided by these frameworks, this review synthesizes evidence by organizing AI-assisted music therapy tools into functional components spanning sensing/monitoring, selection/adaptation (recommendation and generation), and user engagement/feedback, and evaluates how these components map onto therapeutic aims, outcomes, and implementation constraints.

## 2 Methods

### 2.1 Review design

This article is a narrative review synthesizing research and representative tools on AI-assisted music therapy for mental health interventions. A narrative approach was adopted to integrate interdisciplinary evidence (psychology, music therapy, and AI) and to summarize functional pathways, application contexts, and implementation challenges in a rapidly evolving and methodologically heterogeneous field.

### 2.2 Literature identification and data sources

To enhance transparency, we conducted a targeted literature search in PubMed, PsycINFO, Web of Science, IEEE Xplore, Scopus covering publications from 2008 to 2025. Search terms combined AI-related keywords (e.g., “artificial intelligence,” “machine learning,” “deep learning,” “transformer,” “GPT,” “emotion recognition,” “biofeedback”) with music-therapy and mental-health terms (e.g., “music therapy,” “music intervention,” “mental health,” “depression,” “anxiety,” “stress,” “sleep,” “emotion regulation,” “dementia,” “autism”). Reference lists of included papers were also screened to identify additional relevant studies. To position this review and verify originality, we additionally conducted a targeted redundancy check in Google Scholar and Scopus to identify recent reviews with overlapping scope (using combinations such as “AI-assisted music therapy,” “generative music” AND “mental health,” “biofeedback” AND “music intervention,” and “music therapy” AND “machine learning”).

### 2.3 Inclusion focus and selection process

We prioritized studies and technical/clinical reports that (a) described AI-assisted music therapy tools or interventions with clear links to mental health aims, and/or (b) reported evaluation outcomes (e.g., symptom change, feasibility/acceptability, engagement, or system performance used for therapeutic adaptation). We excluded purely conceptual commentary without described intervention logic, purely algorithmic music-generation work without a mental health context, and product marketing materials lacking methodological description. Because this is a narrative review, study selection emphasized relevance, representativeness, and methodological clarity rather than exhaustive coverage.

### 2.4 Data extraction and organization framework

For each included study/tool, we extracted key information such as publication year, country/setting, target population, mental health outcomes, intervention format (e.g., mobile/wearable/clinic-based), AI functions, and main findings. Evidence was organized using a functional framework spanning (i) sensing/monitoring, (ii) selection and adaptive adjustment (recommendation/personalization), (iii) music generation, and (iv) user feedback/engagement, and then mapped to application scenarios and implementation constraints.

In addition, we coded the AI techniques reported in each study/tool using a pragmatic taxonomy: (a) sensing/recognition models (e.g., CNN-based facial expression analysis; audio-feature models for speech/emotion; text sentiment classifiers; multimodal fusion), (b) temporal/physiological modeling (e.g., RNN/LSTM/temporal CNN for HR/EDA/EEG streams), (c) recommendation/adaptive control (e.g., rule-based logic, supervised learning, contextual bandits/reinforcement learning where reported), and (d) generative music models (e.g., Transformer-based sequence models and GPT-style architectures for symbolic/audio generation, alongside rule-based or evolutionary approaches). We extracted these labels only when explicitly described by the source; otherwise, the technique was recorded as “not specified.”

Where studies reported predictive modeling, we extracted whether a train/validation/test split or cross-validation strategy was used, the prediction target (classification/regression), and the evaluation metrics (e.g., accuracy, AUC, F1, RMSE). When such information was not reported, it was coded as “not reported” and treated as a limitation when interpreting evidence strength.

### 2.5 Synthesis approach and evidence considerations

Findings were synthesized qualitatively using narrative synthesis. Given heterogeneity in designs and outcomes, no meta-analysis was conducted. We did not perform a formal risk-of-bias appraisal using standardized tools; instead, we reported study design features (e.g., sample size, comparator presence, follow-up duration) and highlighted common limitations (e.g., small samples, proof-of-concept evaluations, limited external validation) when interpreting the strength and generalizability of evidence.

### 2.6 Case selection criteria

Representative systems were selected using reproducible criteria: (1) public availability and sufficient technical documentation to allow verification; (2) clear mapping to at least two modules of the proposed closed-loop framework (sensing/assessment, personalization/adaptive control, generative approaches, and/or deployment/remote support); (3) the presence of at least one form of publicly reported evaluation (peer-reviewed study, pilot/feasibility report, or documented clinical/real-world deployment outcomes); and (4) coverage of distinct application contexts (e.g., psychotherapy-oriented adaptive music, daily functional soundscapes, and algorithmic composition for relaxation/clinical settings). Wavepaths, Endel, and Melomics-Health were included because each meets these criteria while representing different illustrative implementation pathways.

## 3 Findings

To avoid conflating technologies with therapeutic interventions, we distinguish (i) technology components (sensing/assessment, inference, personalization, generation, delivery/logging) from (ii) intervention characteristics (target population, therapeutic aim, delivery format, dose/duration, comparator, outcomes, and evidence level). We compare representative tools/studies using a consistent set of dimensions, including inputs/signals, core AI function, music output, integration into therapeutic workflow, target outcomes, and evaluation rigor (e.g., feasibility vs controlled evidence).

We highlight the limitations in current evaluations, such as small sample sizes, limited follow-up durations, and inconsistent outcome measures, which hinder the generalizability of the findings. These limitations underscore the need for more systematic, controlled studies with larger and more diverse sample populations to draw stronger conclusions about the clinical effectiveness of AI-assisted music therapy.

### 3.1 Functional architecture of AI-assisted music therapy tools

**FIGURE 1:**
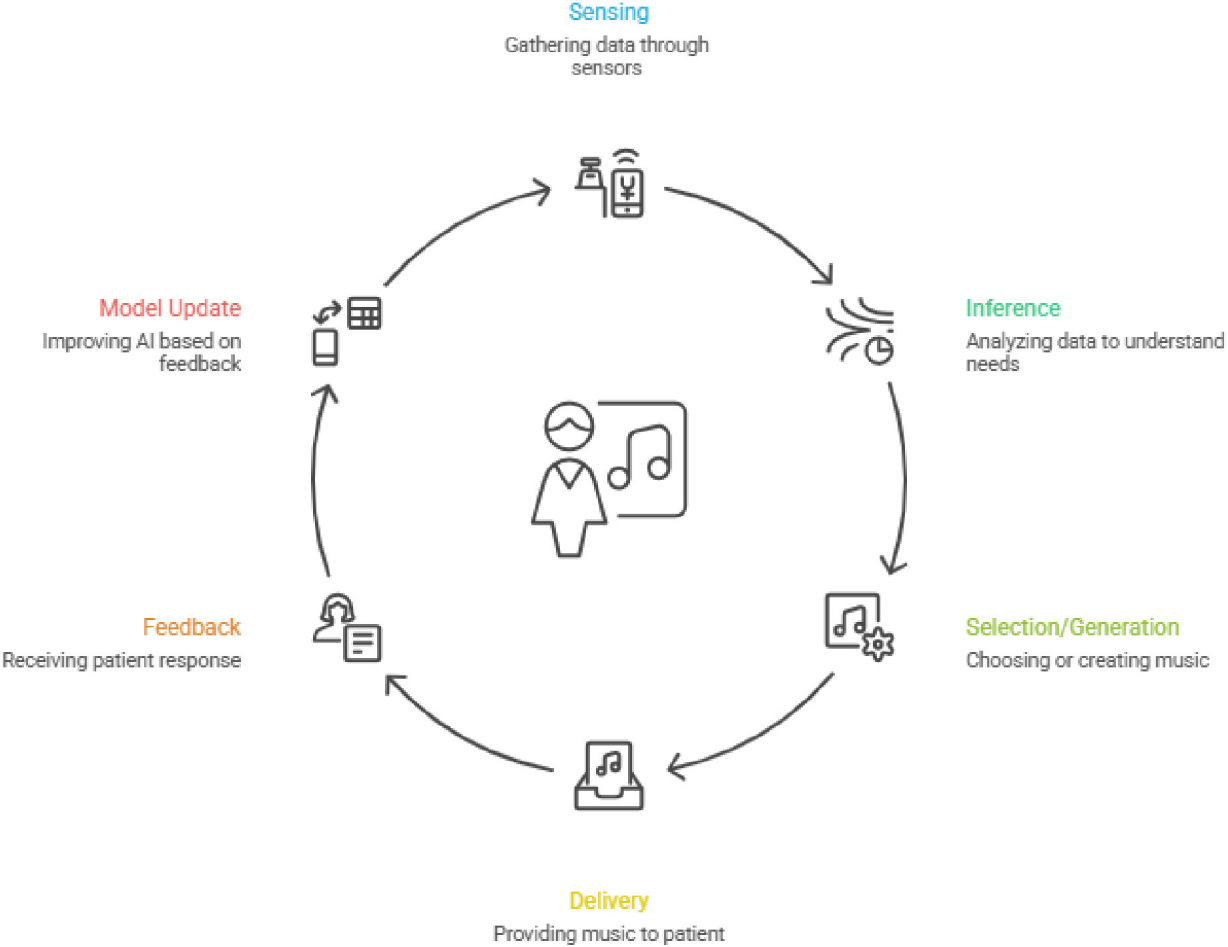
AI-assisted music therapy cycle.

Across the literature, AI-assisted music therapy tools are commonly described as a closed-loop architecture that links technical foundations (e.g., affective computing, recommendation algorithms, generative models, and biofeedback sensing) with user-facing therapeutic functions (e.g., monitoring, content adaptation, feedback, and remote delivery). In general, these systems operate through a sequential and iterative pipeline (Figure 1): (1) sensing, in which multimodal signals such as facial expressions, voice features, text inputs, and physiological indicators (e.g., heart rate, electrodermal activity, EEG) are captured; (2) inference, whereby AI models estimate the user’s current affective state or therapeutic needs from these inputs; (3) selection/generation, in which the system recommends, adapts, or generates music content aligned with the inferred state and intervention goal; (4) delivery, where music is delivered via mobile or cloud-based platforms in real-world settings; (5) feedback, combining passive response signals and active self-reports to evaluate immediate effects; and (6) update, where the system adjusts parameters, refines user profiles, and optimizes subsequent music prescriptions over repeated sessions. This functional architecture provides the organizing logic for the following sections, which synthesize evidence by key system components spanning sensing/monitoring, personalization and adaptive adjustment, generative music, and deployment/engagement features.

### 3.2 Sensing and assessment: emotion recognition and physiological biofeedback

A central enabling layer of AI-assisted music therapy tools is sensing and assessment, in which systems monitor users’ affective states using multimodal signals and translate these inputs into intervention decisions. In practice, monitoring commonly integrates facial cues (e.g., expression dynamics captured by cameras), voice cues (e.g., prosody and tonal variation captured by microphones), and text cues (e.g., sentiment signals from user inputs), together with physiological indicators such as heart rate (HR), electrodermal activity (EDA), blood oxygen saturation, and, in some settings, electroencephalography (EEG) (Son and Jeong, 2025). These signals provide a data-driven basis for estimating whether a user is relaxed, anxious, or depressed, and they are used to guide downstream decisions such as music selection, parameter adjustment, and safety-oriented modulation during sessions.

Representative implementations illustrate how this sensing layer enables closed-loop adaptation. For example, Shin et al. (2014) described a stress-relief music system in which a miniature finger-worn sensor continuously measured heartbeats and transmitted the data wirelessly for stress estimation; the system then used heart-rate variation as an index of tension and adapted music recommendations in real time to reduce physiological stress. In applied dementia care, the Resonance Rx system uses facial-expression mapping to detect emotional responses during listening and adjusts playlists accordingly, aiming to alleviate anxiety and agitation in patients (LUCID, 2025).

However, the current evidence base also highlights recurring constraints: emotion inference accuracy can vary across contexts and individuals, multimodal models may show limited generalizability across age groups and clinical populations, and continuous monitoring raises practical and ethical concerns regarding privacy, consent, and data security—particularly when cameras or sensitive physiological signals are involved.

### 3.3 Personalization and adaptive adjustment: recommendation, control, and user feedback

Beyond sensing, most AI-assisted music therapy tools are defined by a personalization and adaptive control layer that translates assessed states and user profiles into individualized “music prescriptions.” The decision logic typically combines (i) rule-based strategies (e.g., therapist-defined constraints or heuristic mappings from states to musical properties), (ii) machine-learning–driven personalization using historical preferences and response data, and in some systems (iii) closed-loop adaptive tuning in which parameters are iteratively adjusted based on real-time signals and accumulated user feedback. Personalized recommendation algorithms operationalize this logic by integrating factors such as prior listening preferences, current affective state, and therapeutic goals to select or tailor musical content aligned with the user’s psychological needs; for example, data mining–based action rule frameworks have been applied to guide therapeutic music selection, linking recommendation outputs to treatment objectives (Benedict et al., 2025).

A key feature of this layer is the explicit coupling of triggers and adaptation strategies. In many implementations, changes in inferred state (e.g., rising tension, reduced engagement, or deteriorating mood) serve as triggers that prompt the system to adjust content. The adjustment strategy may involve switching tracks, modifying musical parameters (e.g., tempo, intensity, instrumentation, or emotional tone), or smoothly transitioning between soundscapes to avoid abrupt stimulation. As an illustrative implementation pathway, Wavepaths provides real-time music adaptation within therapist-defined boundaries, allowing musical elements to be modulated dynamically in response to user state cues (Wavepaths, 2025). Similarly, Endel generates context-aware soundscapes and adapts output based on environmental and optional physiological inputs, illustrating how personalization can be delivered at scale in daily-life settings (Zenora, 2025).

User feedback mechanisms further close the personalization loop by incorporating active self-report (e.g., preference ratings, perceived relaxation, mood logs, or brief post-session questionnaires) alongside passive signals to update user profiles and refine subsequent recommendations (Zenora, 2025). This bidirectional interaction can enhance perceived autonomy and engagement while providing the system with data to iteratively improve alignment between user preferences and therapeutic aims. At the same time, available descriptions and evaluations remain heterogeneous, and for many commercial platforms, evidence is often limited to feasibility, usage patterns, or preliminary reports rather than controlled outcome studies, underscoring the need for more rigorous comparative evaluations of adaptive strategies and their clinical impact.

### 3.4 Generative music approaches for therapeutic delivery

A distinctive pathway in AI-assisted music therapy involves generative music approaches, in which models are used not merely to select from existing libraries but to generate, extend, or stylistically adapt musical material in ways that align with targeted therapeutic goals (e.g., relaxation, anxiety reduction, or mood modulation). In therapeutic contexts, generative models are typically positioned as a mechanism for producing on-demand, personalized content, enabling continuous delivery and fine-grained control over musical features such as tempo, harmonic stability, timbral density, and dynamic contour.

Recent work emphasizes deep learning–based generative architectures—particularly Transformer and GPT-style models—which can learn structural and stylistic regularities from large-scale music corpora and then compose or extend musical sequences in a more controllable manner (Banar and Colton, 2022; Jin et al., 2020). Alongside these data-driven architectures, alternative paradigms remain influential, including evolutionary and rule-based algorithms that explicitly encode compositional constraints and optimization objectives. A prominent example is the Melomics system, which employs bio-inspired algorithms for autonomous composition; its clinical derivative, Melomics-Health, applies algorithmic composition principles to generate music intended to promote relaxation and psychological well-being (Raglio and Vico, 2017).

Evidence from Melomics-Health illustrates how algorithmic composition can be operationalized as “customizable acoustic medicine,” with musical parameters designed to minimize abrupt changes and support sustained calm. In clinical and perioperative settings, Melomics-generated relaxation music has been reported to alleviate anxiety and pain, suggesting the feasibility of using AI-composed material as a therapeutic stimulus (Requena et al., 2014). Comparative work has further suggested that algorithmically generated relaxation music may produce relaxation responses that are comparable to human-composed music on both self-report and physiological indicators (e.g., skin conductance, heart rate), challenging the assumption that machine-generated music is inherently less effective for emotional regulation (Raglio et al., 2021).

At the same time, key evidence gaps remain. Many studies evaluate generative music primarily through short-term outcomes or constrained experimental settings, with limited head-to-head comparisons against established interventions and insufficient evidence on durability, individual differences, and real-world adherence. Moreover, deep generative models raise additional concerns regarding explainability (i.e., why specific generated features should be expected to influence a given psychological mechanism), content safety and quality control (e.g., avoiding unsettling musical trajectories or unintended emotional effects), and governance of training data and personalization. These issues underscore the need for more rigorous, mechanism-informed evaluation frameworks before generative music can be considered a mature clinical component in AI-assisted music therapy.

### 3.5 Remote delivery, logging, and data visualization

A further layer shaping the real-world feasibility of AI-assisted music therapy tools is the deployment infrastructure that enables remote delivery, session logging, and outcome monitoring over time. In many systems, interventions are delivered through mobile applications or cloud-based platforms, allowing users to access music-based support via smartphones, tablets, or wearable devices across home, workplace, or community settings. This shift toward mobile and teletherapeutic delivery can expand reach beyond clinic-based services and supports continuity of care between in-person encounters by embedding therapeutic routines into everyday life. Endel, for example, illustrates how personalized soundscapes can be delivered in daily contexts, while optional remote monitoring features allow progress to be reviewed outside the clinic (Zenora, 2025).

In parallel, digital platforms typically implement structured intervention recording and data visualization. Systems can log session-level data such as musical selections, listening duration, physiological and emotional responses, and user feedback, and then summarize these data through dashboards for clinicians and users (Zenora, 2025). Visualization formats often include time-series trends in mood ratings, physiological indices aligned with musical features, and session-to-session changes that can support therapeutic decision-making and facilitate follow-up planning. Beyond individual care, aggregated and anonymized logs may also provide a resource for examining associations between musical characteristics and outcomes, thereby informing iterative improvement of tool design and evaluation.

However, deployment at scale also introduces practical constraints that may influence effectiveness, including variability in user engagement and adherence, differences in digital literacy and device access, and the potential for a “digital divide” to exacerbate inequities in who benefits from remote interventions. These considerations highlight that accessibility gains from mobile delivery are accompanied by new implementation challenges that require attention in both evaluation design and ethical governance.

### 3.6 Representative systems as illustrative cases

To illustrate how the above functional architecture is implemented in practice, this section summarizes several representative systems as illustrative implementation pathways (Table 1). Rather than treating these platforms as definitive evidence of clinical efficacy, we use them to exemplify distinct design logics (e.g., adaptive personalization, context-aware soundscapes, and algorithmic composition) and to highlight the current state of evaluation in real-world deployments.

**TABLE 1:** Comparative overview of illustrative AI-assisted music therapy systems and their reported evaluation evidence.

Comparison dimension	Wavepaths	Endel	Melomics-health
Primary use case and target population	Psychotherapy-oriented emotional processing; adults in therapy / mental wellness users	Everyday mental wellness (sleep/focus/stress); general population	Relaxation/clinical adjunct (e.g., perioperative anxiety/pain; stress reduction)
Delivery setting	Clinic/therapist-guided sessions; potentially remote wellness use	Mobile app / consumer platform	Clinical settings and structured listening sessions
Inputs for sensing/assessment	User feedback during sessions; optional physiological/behavioral cues as reported	Context signals (time/location/activity), optional wearable data, self-report (depending on implementation)	Typically not sensing-heavy; may rely on protocolized delivery and outcomes
Core AI technologies	Adaptive control logic; generative/adaptive music engine; personalization rules (exact architecture often not fully disclosed in public docs)	Context-aware personalization; adaptive soundscape generation; recommendation/parameter tuning (specific models not always disclosed)	Algorithmic composition (rule-based/evolutionary/bio-inspired), parameterized generation of “therapeutic” music
Music/intervention approach	Continuous adaptive soundscapes to support affect regulation and therapeutic process	Algorithmic soundscapes aimed at relaxation/focus rather than clinical therapy *per se*	Generates unfamiliar, parameter-controlled music intended to induce relaxation
Adaptation mechanism	Uses state cues → adjust parameters (tempo/intensity/timbre) and session-phase constraints; feedback-informed personalization	Context/physiology → generate/adjust soundscape; iterative personalization via user behavior	Primarily pre-generated or protocol-driven generation; limited real-time sensing in many uses
Evidence of efficacy / evaluation	Reported early-stage/pilot evaluation and/or usage reports (depending on source availability)	Predominantly real-world usage/feasibility and limited research evidence (varies by publication/partner reports)	Peer-reviewed clinical/pilot studies reported in some contexts (e.g., anxiety/pain outcomes)
Key limitations to note	Public evidence may be heterogeneous; often limited details on study design, comparators, and long-term outcomes	Often not disorder-specific; clinical endpoints and controlled trials may be limited; risk of overgeneralizing wellness outcomes to clinical efficacy	Generalizability across disorders/populations uncertain; mechanism and personalization depth varies; limited head-to-head comparisons with therapist-selected music

#### 3.6.1 Wavepaths (adaptive personalization in psychotherapy)

Wavepaths exemplifies a psychotherapy-oriented pathway in which generative or adaptive music is embedded within therapist-guided contexts. Publicly available descriptions indicate that the platform supports real-time modulation of musical elements within therapist-defined constraints, aligning music delivery with the user’s moment-to-moment state and therapeutic intent (Wavepaths, 2025). However, it is important to note that the current evidence base for Wavepaths relies largely on early-stage implementation reports and platform descriptions, with limited controlled clinical evaluations. Further, more rigorous, systematic outcome studies are required to validate its clinical effects. Conceptually, Wavepaths maps closely onto a closed-loop model: state cues and therapist inputs inform adaptive adjustment (e.g., intensity, pacing, or soundscape progression), and the resulting outputs are iteratively updated during sessions. However, the current evidence base appears to rely largely on early-stage implementation reports and platform descriptions, with limited publicly available controlled evaluations, underscoring the need for more systematic outcome studies in clinical psychotherapy settings.

#### 3.6.2 Endel (soundscape-based daily mental health support)

Endel represents a consumer-facing pathway focused on daily-life mental health support through context-aware soundscapes. However, as with other commercially available systems, the current evidence for Endel is largely based on real-world usage reports and feasibility studies. These studies often lack controlled clinical trials and standardized outcome measures, making it difficult to draw definitive conclusions about its clinical efficacy. More controlled studies are needed to confirm the therapeutic effects of these soundscapes in diverse populations. The system is described as integrating contextual and optional physiological inputs to generate or adapt ambient sound environments aimed at supporting relaxation, focus, and sleep in naturalistic settings (Zenora, 2025). From an implementation perspective, Endel illustrates how personalization can be operationalized at scale via mobile delivery and continuous adaptation, prioritizing accessibility and routine integration. At the same time, evaluation evidence for many consumer wellness applications is often heterogeneous, and reported benefits may not be grounded in clinical-grade study designs, highlighting the importance of transparent claims, standardized outcome measures, and independent validation.

#### 3.6.3 Melomics-Health (algorithmic composition for relaxation and clinical contexts)

Melomics-Health illustrates an algorithmic composition pathway in which therapeutic music is generated through computational control of musical parameters rather than relying on preexisting libraries. Prior clinical and perioperative applications have reported that algorithmically generated relaxing music can be used to support anxiety and pain management, suggesting feasibility for structured clinical contexts (Requena et al., 2014). Comparative evidence has further suggested that algorithmically generated relaxation music may elicit relaxation responses comparable to human-composed music on both subjective and physiological indicators in controlled settings (Raglio et al., 2021). As an illustrative case, Melomics-Health demonstrates how “parameterized” composition can be designed around intended physiological or affective targets; nevertheless, broader clinical generalizability, long-term outcomes, and differential effects across populations remain underexplored.

#### 3.6.4 Special populations (dementia and cognitive impairment)

Across dementia and cognitive-impairment contexts, AI-assisted music therapy tools are often positioned as scalable mechanisms for tailoring familiar or calming music and for supporting behavioral and emotional regulation. Systems described in this space typically emphasize personalization (e.g., age cohort, cultural background, and listening history) and adaptive updating based on user responses, aiming to reduce agitation, support mood, and encourage engagement. Related clinical literature suggests that music-based interventions can improve emotional well-being and behavioral symptoms in dementia, providing a rationale for AI-enabled personalization at scale (Raglio and Vico, 2017). However, much of the AI-specific evidence remains preliminary, with a need for clearer reporting of study designs, independent evaluations in care settings, and careful attention to consent, privacy, and caregiver-mediated deployment.

## 4 Discussion

### 4.1 Summary of main findings

Across the reviewed literature and illustrative systems, AI-assisted music therapy tools converge on a relatively consistent functional architecture centered on (i) sensing and assessment (multimodal emotion recognition and/or physiological monitoring), (ii) personalization and adaptive control (recommendation, parameter tuning, and closed-loop adjustment), (iii) generative content pathways (algorithmic or deep generative music creation), and (iv) deployment and engagement infrastructure (mobile delivery, session logging, and visualization for follow-up). In practice, many systems prioritize state monitoring + adaptive content delivery, reflecting a design logic in which music is treated as a modifiable stimulus whose features can be aligned with momentary affect and therapeutic phase.

However, the evidentiary strength across modules remains uneven. Reports and early evaluations are most commonly available for monitoring and personalization functions (e.g., inferring state and adapting playlists/soundscapes), whereas generative music approaches are less consistently evaluated in controlled clinical contexts despite their conceptual appeal and technical feasibility. Across domains, the most recurrent limitations include small samples, heterogeneous outcome measures, short follow-up windows, and limited use of controlled comparisons—factors that constrain causal inference and make it difficult to determine which system components are responsible for observed benefits. These shared gaps also complicate cross-study synthesis and hinder translation into standardized clinical pathways.

### 4.2 Interpretation through theoretical frameworks

The observed architecture is theoretically coherent when mapped to core psychological and neurobehavioral frameworks. From an emotion regulation perspective, the emphasis on sensing, state inference, and adaptive music delivery aligns with the premise that regulation is facilitated when strategies are matched to current affective demands (e.g., down-regulating arousal during anxiety or supporting activation during low mood). In this view, personalization is not simply preference matching; rather, it operationalizes a regulation hypothesis—namely, that music selection and parameter adjustment can function as a controllable regulatory strategy by shaping attention, appraisal, and affective intensity in context.

From a neuroscientific perspective, the focus on reward-, attention-, and communication-related components provides a plausible mechanistic rationale for why certain musical features (e.g., rhythmic entrainment, pleasantness, predictability) may influence mood and stress physiology. Systems that adapt tempo, intensity, or harmonic stability can be interpreted as attempts to target neural circuits associated with reward and affect, to support attentional modulation, and—when used in interpersonal or guided settings—to scaffold social engagement and communication. At the same time, mechanistic claims often outpace direct evidence, because many studies lack neural measures or do not explicitly test mechanism-linked hypotheses.

Finally, human–computer interaction (HCI) theory clarifies why engagement-oriented features—user control, feedback loops, usability, and transparent interaction—are not peripheral design choices but core determinants of adherence and perceived efficacy. Tools that enable user feedback, autonomy, and iterative co-adaptation may enhance motivation and sustained use, whereas opaque algorithms or overly automated experiences may reduce trust or increase drop-out. In short, the theoretical frameworks collectively suggest that effectiveness depends not only on “having AI,” but on whether AI is integrated in ways that support state-appropriate regulation, mechanism-aligned stimulus design, and human-centered engagement.

### 4.3 Challenges and limitations

#### 4.3.1 Individual differences and precision adaptation

Individual variability remains a primary barrier to robust personalization. Musical preferences and psychological responses differ markedly across users and contexts, and many current models are trained on general datasets that may not adequately represent subgroups (e.g., adolescents, older adults, culturally diverse users). Moreover, both short-term affective fluctuations and long-term preference drift create moving targets for adaptation; without sufficient longitudinal data and context modeling, systems risk delivering mismatched content that undermines therapeutic aims.

#### 4.3.2 Ethical, privacy, and governance risks

AI-assisted music therapy often relies on sensitive data streams (e.g., facial images, voice, HR/EDA, EEG), raising substantial privacy and governance concerns. Risks include insecure data handling, secondary use beyond therapeutic purpose, and inequitable performance due to dataset bias. In addition, the ethical legitimacy of AI-mediated care warrants careful framing: highly automated interventions may dilute humanistic elements of mental health support if they are deployed as substitutes rather than complements to professional care and social support.

#### 4.3.3 Outcome evaluation and evidence gaps

Outcome evaluation remains fragmented. Music-therapy-relevant outcomes are frequently subjective and context-sensitive, and many AI-enabled tools rely on self-report or limited physiological indices without standardized endpoints, adequate controls, or long-term follow-up. Commercial products may emphasize engagement metrics that are not equivalent to clinical benefit. Collectively, these issues weaken scientific credibility and impede meta-level synthesis, making it difficult to compare tools, isolate active ingredients, or define minimum evidence thresholds for deployment.

#### 4.3.4 Therapy dependence and self-efficacy concerns

A further risk is unintended dependence on automated soothing or regulation. If users repeatedly rely on AI-generated music as a default coping response, self-regulatory capacity and confidence may erode, particularly among adolescents whose self-control skills are still developing. Additionally, an exclusively self-guided model may reduce interpersonal interaction that is often central to therapeutic change, potentially exacerbating isolation in vulnerable individuals.

#### 4.3.5 Limitations of this narrative review

This review is narrative in nature and therefore not exhaustive. Although the literature selection process was structured, narrative synthesis is inherently more vulnerable to selection bias, variability in reporting quality, and heterogeneity in study designs and outcomes. Furthermore, the rapidly evolving commercial landscape means that product capabilities may change faster than peer-reviewed evaluation, limiting the stability of inferences drawn from platform descriptions or early-stage reports.

### 4.4 Practical implications and optimization strategies

#### 4.4.1 Improving precision and adaptability

A pragmatic roadmap for improving personalization is to move from “generic personalization” to context-sensitive precision adaptation. This includes richer user profiling (age, cultural background, symptom profiles), segmentation models for subgroup tailoring, and explicit incorporation of contextual variables (time of day, setting, social context). Systems should also support human–AI co-adaptation by combining algorithmic inference with user-adjustable controls and feedback, enabling iterative tuning when automated decisions are mismatched.

#### 4.4.2 Privacy-by-design and ethical safeguards

Ethical implementation requires privacy-by-design: data minimization, secure local processing when feasible, strong encryption for transmission and storage, and transparent consent pathways. Governance should include regular bias checks, documentation of model limitations, and clear user-facing disclosures regarding data usage and algorithmic logic. For higher-stakes deployments (clinical settings, dementia care), third-party audits and compliance with professional standards should be treated as baseline requirements rather than optional add-ons.

#### 4.4.3 Evaluation standards and clinical translation pathways

To strengthen credibility and clinical usefulness, the field needs convergent evaluation standards. Comparative designs (including RCTs where feasible) should test adaptive AI music against appropriate comparators (standard care, non-AI music interventions, or matched attention controls). A minimum evaluation set should include validated symptom scales (e.g., anxiety/depression), adherence metrics, and mechanism-relevant indicators (e.g., HRV or stress biomarkers when appropriate), alongside longer-term follow-up. Translational pathways should clarify target populations, contraindications, and integration models with clinician oversight rather than treating tools as universally applicable.

#### 4.4.4 Supporting autonomy and self-regulation

To reduce dependence risk, systems should be designed as assistive rather than substitutive. Practical options include built-in psychoeducation, prompts for diversified coping strategies, and usage moderation features that flag excessive reliance. Where appropriate, social and group-based elements can reintroduce interpersonal support and reduce isolation. Clinicians can also guide users from passive consumption toward active co-selection or co-creation of music to strengthen self-efficacy and engagement.

### 4.5 Conclusions and future directions

Overall, current evidence suggests that AI-assisted music therapy tools are most mature as scalable, adaptive delivery systems that combine monitoring, personalization, and mobile deployment to support emotion regulation and mental wellness in real-world settings. Synthesizing the reviewed studies and representative tools, the evidence converges on a closed-loop functional architecture—sensing/assessment, personalization/adaptive control, content generation or tailoring, and deployment/logging—while the distribution and strength of evidence remain uneven across modules and outcomes. At the same time, the field cannot yet make uniform, high-confidence claims about clinical efficacy across disorders and populations, because controlled evidence remains heterogeneous and often limited by small samples, inconsistent endpoints, and short follow-up. Accordingly, AI-assisted music therapy should be framed as a promising, rapidly developing adjunct rather than a replacement for established clinical care. Practically, stakeholders should prioritize state–music matching, report intervention dosage and adherence, and adopt privacy-by-design safeguards for multimodal sensing to support safe real-world implementation.

Future research should prioritize (i) rigorous comparative trials with clearly defined comparators and standardized outcomes, (ii) external validation across cultures, age groups, and clinical subpopulations, (iii) multimodal and longitudinal monitoring to evaluate durability and individual trajectories, and (iv) mechanism-informed designs that explicitly test how musical features and adaptive control strategies relate to emotion regulation processes and neurophysiological markers. In parallel, the development agenda should include privacy-by-design governance, transparent reporting standards, and clinically meaningful evaluation benchmarks, ensuring that technological innovation translates into safe, equitable, and genuinely human-centered mental health support. To strengthen reproducibility, future work should also report AI components and model evaluation practices (e.g., train/validation/test splits or cross-validation where applicable), enabling clearer comparisons across systems and studies.
